# Factors Associated With Chinese Adults’ Vaccine Acceptance

**DOI:** 10.1001/jamahealthforum.2021.1466

**Published:** 2021-07-09

**Authors:** Haiyang Yang, Jingjing Ma

**Affiliations:** 1 Johns Hopkins University, Baltimore, Maryland; 2 Peking University, Beijing, China

## Abstract

This survey study assesses disparities in COVID-19 vaccine acceptance and identifies approaches to improve vaccination rates among adults in China.

## Introduction

Turning COVID-19 vaccines into vaccinations is a critical step to ending the COVID-19 pandemic.^1,2^ It is thus imperative to understand the disparities in COVID-19 vaccine acceptance and identify approaches to improve vaccination rates. The aim of this study was to add to this understanding.

## Methods

This survey study received institutional review board clearance from Johns Hopkins University as well as approval by the National School of Development at Peking University where the data collection took place. The data were collected in January 2021, by which time the nationwide distribution of COVID-19 vaccines had just begun in China. A large-scale, nationally representative, random sample of 14 378 Chinese adults were invited to participate in this internet-based survey. The survey was conducted following the guidelines by the American Association for Public Opinion Research (AAPOR). Participants consented before responding to the questionnaire, responded anonymously, and could terminate their participation at any point (eMethods 1 in the Supplement).

Participants indicated whether they had been vaccinated for COVID-19. Those who had not been vaccinated indicated their willingness to receive the vaccine. Participants then rated their knowledge about COVID-19 vaccines on 4 scales (eMethods 2 in the Supplement).

Next, we assessed how information about the vaccination behavior of the general public vs socially proximal others might be associated with vaccination decision-making. Participants who indicated that they were not yet willing to receive the vaccine were randomly assigned to respond to 1 of 2 versions of an additional question: one-half indicated what percentage of the general public had to be vaccinated for COVID-19 before they themselves would be vaccinated; for the other half, the reference group was “people you personally know.” All participants then completed sociodemographic measures.

Responses to the vaccine knowledge items were averaged into a single measure (α = .73). A dummy variable was created to represent whether participants resided in Hubei, the province where COVID-19 was first found in China.^3^ Ordinary least squares regression and mediation analyses (eMethods 3 in the Supplement) were conducted using SAS, version 9.4 (SAS Institute) to examine the associations between vaccination measures and sociodemographic variables.

## Results

A total of 12 651 participants (6145 [48.6%] women; mean age, 36.6 years; 7715 [61.0%] married; from 32 provincial regions) completed the study (88% response rate). Only 2% of participants were already vaccinated for COVID-19. Among the rest, 1.3% indicated that they definitely would not receive the vaccine, 3.5% probably would not, 9.1% were uncertain, 37% probably would, and 47% definitely would (see Table 1 for more details). An ordinary least squares regression revealed that women and individuals with lower incomes or education levels were less likely to indicate that they would receive the vaccine (Table 2). Higher vaccine knowledge ratings were associated with higher willingness to be vaccinated (*r* = 0.31; *P* < .001). Mediation analyses showed that knowledge ratings significantly mediated differences in vaccine acceptance across income and education levels but not gender.

**Table 1.** Descriptive Statistics (n = 12 651)

Characteristic	No. (% in category or mean of variable)^a^	Mean of willingness to be vaccinated^b^	Willing to be vaccinated, %^c^
Age, mean (SD), y	12 649 (mean [SD], 36.6 [14.6])	NA	NA
Gender			
Women	6145 (48.6)	4.2	84.4
Men	6504 (51.4)	4.3	87.2
Monthly household income, mean (SD), ¥10 000	11 876 (mean [SD], 1.7 [1.6])	NA	NA
Education			
With college degree	9484 (75.0)	4.3	86.8
Without college degree	3162 (25.0)	4.2	83.0
Marital status			
Married	7715 (61.0)	4.3	85.9
Not married	4936 (39.0)	4.3	85.8
Location			
Rural	1382 (10.9)	4.3	85.8
Urban	11 269 (89.1)	4.3	85.9
Province			
Hubei	631 (5.0)	4.3	89.2
Other	12 020 (95.0)	4.3	85.7

Abbreviation: NA, not applicable.

^a^ The number of valid responses.

^b^ Responses were coded as willingness to receive the COVID-19 vaccine (1 = definitely will not; 2 = probably will not; 3 = not sure; 4 = probably will; 5 = definitely will).

^c^ The respective percentage of participants who selected either “definitely will” or “probably will” on the COVID-19 vaccine acceptance measure.

**Table 2.** Disparities of COVID-19 Vaccine Acceptance Across Sociodemographic Segments

Variable	Association with willingness to receive COVID-19 vaccine^a^		Vaccine knowledge as a mediator of the association^b^			
	β (SE)	*P* value	Direct effect sizes		Indirect effect size via knowledge	
			β (SE)	95% CI	β (SE)	95% CI
Gender, 1 = woman, 0 = man	−0.075 (0.014)	<.001	−0.076 (0.015)	−0.106 to −0.046	−0.008 (0.005)	−0.018 to 0.002
Monthly household income, ¥10 000	0.024 (0.005)	<.001	0.005 (0.005)	−0.005 to 0.015	0.016 (0.002)	0.013 to 0.020
Education, 1 = with college degree, 0 = without college degree	0.022 (0.009)	<.02	0.006 (0.011)	−0.015 to 0.026	0.038 (0.004)	0.032 to 0.046
Marital status, 1 = married, 0 = not	0.003 (0.021)	.88	NA	NA	NA	NA
Location, 1 = urban, 0 = rural	−0.035 (0.025)	.17	NA	NA	NA	NA
Age, y^c^	−0.001 (0.001)	.10	NA	NA	NA	NA
Hubei, 1 = yes, 0 = no	0.023 (0.033)	.48	NA	NA	NA	NA
Intercept	4.329 (0.058)	<.001	NA	NA	NA	NA

Abbreviation: NA, not applicable.

^a^ Results of ordinary least squares regression.

^b^ Results of 3 bootstrap mediation analyses. Gender, income, or education was used respectively as the independent variable, vaccine knowledge ratings as the mediator, and willingness to vaccinate as the dependent variable. The other sociodemographic variables were included as covariates.

^c^ Age was a continuous variable, which was included in the ordinary least squares regression along with the other continuous (eg, income) and categorical (eg, gender) variables as the predictors.

Those not yet willing to be vaccinated indicated that they would receive the vaccine when a mean of 64% (SD, 22%) of the general public had been vaccinated. This ratio was significantly lower if the reference group was “people you personally know” (54%; SD, 29.9%; *F*_1,1539_ = 67.67; *P* < .001).

## Discussion

In this survey study of Chinese adults, we found that women and individuals with lower incomes or education levels indicated that they were less willing to be vaccinated. Vaccine knowledge mediated the latter 2 associations. Individuals who were not yet willing to receive the vaccine took account of vaccination behaviors of others, particularly socially proximal others, in deciding when they would receive vaccination.

Adding to a growing stream of research,^4,5^ these findings highlight the need to address disparities in COVID-19 vaccine acceptance across demographic segments and suggest that health policies for improving vaccination should consider leveraging vaccine knowledge dissemination and social influence. Finally, our research focused on China, the world’s most populous nation. However, the extent to which the results are generalizable to other regions requires further investigation.

## Notes

Supplement. **eMethods 1.** Sampling Method **eMethods 2.** Survey Instruments **eMethods 3.** Statistical Analyses
eReferences.
