# Supplementary material for: Factors Associated With Chinese Adults’ Vaccine Acceptance
Source: JAMA Health Forum. 2021 Jul 9;2(7):e211466. doi: 10.1001/jamahealthforum.2021.1466 (PMC8796971; doi:10.1001/jamahealthforum.2021.1466)
Supplement: Supplement. — eMethods 1. Sampling Method eMethods 2. Survey Instruments eMethods 3. Statistical Analyses eReferences. [file jamahealthforum-e211466-s001.pdf]

Supplemental Online Content

Yang H, Ma J. Factors associated with Chinese adults' vaccine acceptance. *JAMA Health Forum*. 2021;2(7):e211466. doi:10.1001/jamahealthforum.2021.1466

eMethods 1. Sampling Method

eMethods 2. Survey Instruments

eMethods 3. Statistical analyses

eReferences.

This supplemental material has been provided by the authors to give readers additional information about their work.

© 2021 Yang H et al. *JAMA Health Forum.*

eMethods 1. Sampling Method

To recruit a nationally representative sample, we worked with a leading research agency in China. This agency operates an online survey panel with over 4 million respondents across all provincial regions in mainland China. We drew a nationally representative sample based on gender, age, and location criteria. 14,378 qualified adults were randomly drawn from the panel, and were sent a survey link along with the description of the study. See below for a comparison of the key demographic variables of our sample and the population in mainland China.

Profile		Population (mainland China)	Sample
**Region**	Northern	20.51%	20.65%
	Eastern	29.41%	29.42%
	Western	21.82%	21.53%
	Middle	16.13%	16.01%
	Southern	12.13%	12.39%
Sum		100%	100%
**Gender**	Male	51.22%	51.42%
	Female	48.78%	48.58%
Sum		100%	100%
**Age group** (year range)	< 25	33.66%	32.30%
	25-34	14.87%	15.44%
	35-44	18.21%	17.63%
	45-54	13.83%	14.81%
	>54	19.43%	19.82%
Sum		100%	100%

Note: Mainland China regional population statistics are from the National Bureau of Statistics.

© 2021 Yang H et al. *JAMA Health Forum.*

eMethods 2. Survey Instruments

The data collection was solely funded by Peking University. The survey was conducted following the guidelines by AAPOR. Participants were given ample time to review the consent information, and consented before responding to the questionnaire. They responded anonymously, and could terminate their participation at any point. The survey questions included two parts: (1) questions related to COVID-19 vaccination and (2) socio-demographic measures.

(1) Questions related to COVID-19 vaccination

当您所居住的地方开始打疫苗的时候，您会接种新冠病毒疫苗吗？(Responses were coded as: whether the participant had already vaccinated [1=yes 0=no]; willingness to receive the COVID-19 vaccine [1=definitely will not, 2=probably will not, 3=not sure, 4=probably will, 5=definitely will])

○ 绝对不会

○ 可能不会

○ 不确定

○ 可能会

○ 绝对会

○ 我已经接种

请问您对新冠疫苗的以下各个方面有多了解？(Four measures on COVID-19 vaccine knowledge [1=completely not knowledgeable, 5=completely knowledgeable]: overall

© 2021 Yang H et al. *JAMA Health Forum.*

understanding of COVID-19 vaccines, how COVID-19 vaccines work, the types of vaccines available, and differences among the vaccines)

	完全不了解	不太了解	不确定	有些了解	非常了解
总的来说，您对新冠疫苗的了解程度	○	○	○	○	○
新冠疫苗的原理	○	○	○	○	○
新冠疫苗的种类	○	○	○	○	○
各种新冠疫苗的不同点	○	○	○	○	○

Participants who were not yet willing to vaccinate (i.e., those who indicated “绝对不会” [“definitely will not”] or “可能不会” [“probably will not”] or 不确定 [“not sure”] in response to the question “当您所居住的地方开始打疫苗的时候，您会接种新冠病毒疫苗吗？” [“When COVID-19 vaccination begins in your area, will you get vaccinated?”]), were randomly shown one of the following questions:

> 您自己认识的人里至少有多少打了疫苗，您才可能打疫苗？(The percentage of the people the participant personally knew had to be vaccinated for COVID-19 before the participant herself/himself would vaccinate.)
>
> 全中国至少有多少人打了疫苗，您才可能打疫苗？(The percentage of the general public had to be vaccinated for COVID-19 before the participant herself/himself would vaccinate.)

© 2021 Yang H et al. *JAMA Health Forum.*

10%以下

10%-19%

20%-29%

30%-39%

40%-49%

50%-59%

60%-69%

70%-79%

80%-89%

90%以上

不论多少人打，我都不打

(Following an established approach[1], each participant’s response to the percentage range scale was coded as a continuous variable by taking the midpoint of the respective interval. Participants who did not select a percentage, were not included into the percentage analysis.)

© 2021 Yang H et al. *JAMA Health Forum.*

(2) Socio-demographic measures

您的性别为：(Gender [1=woman, 0=man])

○ 男性

○ 女性

您目前的婚姻状况为：(Each participant’s response was coded as a dummy variable: Marital Status [1=married, 0=not])

○ 不愿透露

○ 未婚

○ 已婚

○ 分居

○ 离婚

○ 丧偶

○ 其他 ____

© 2021 Yang H et al. *JAMA Health Forum.*

您的家庭年收入为：（单位：元）(Following an established approach[1], each participant's response to this annual household income scale was coded as a continuous variable by taking the midpoint of the respective income interval when a fixed-range scale item was selected. When an open-range item was chosen, we recoded the response using the lower bound. The value was then divided by twelve to create a monthly household income in ¥10,000)

- ○ 1 万以下
- ○ 1 万-2 万以下
- ○ 2 万-3 万以下
- ○ 3 万-5 万以下
- ○ 5 万-10 万以下
- ○ 10 万-50 万以下
- ○ 50 万-100 万以下
- ○ 100 万及以上

- ○ 不愿透露

© 2021 Yang H et al. *JAMA Health Forum.*

您目前的最高学历为：(Each participant’s education level was coded as a dummy variable: Education [1=with a college degree, 0=without a college degree])

- ◯ 未上过小学
- ◯ 小学
- ◯ 初中
- ◯ 高中/中专/技校
- ◯ 本科/大专
- ◯ 硕士
- ◯ 博士
- ◯ 其他 ____

您的出生年份为：____________ (Each participant’s birth year was coded as Age [years])

您常住的省份为：____________ (Each participant’s resident province was coded as a dummy variable: Hubei [1=yes, 0=no])

您的常住地是：(Locale Type [1=urban, 0=rural])

- ◯ 城镇
- ◯ 农村

© 2021 Yang H et al. *JAMA Health Forum.*

eMethods 3. Statistical Analyses

OLS regressions were used to analyze the associations between socio-demographic variables and vaccine acceptance.

To examine the role of vaccine knowledge in the significant associations between the three socio-demographic variables (Gender, Income, Education) and vaccine acceptance, we conducted three mediation analyses (PROCESS[2]; 5000 bootstrap resamples; Model 4) with each of the three socio-demographic variables (Gender, Income, Education) as the independent variable, vaccine knowledge rating as the mediator, vaccine acceptance as the dependent variable, and the other socio-demographic variables as covariates. The direct effect of each of the three socio-demographic variables on vaccine acceptance and its indirect effect on vaccine acceptance through knowledge are reported in Table 2.

© 2021 Yang H et al. *JAMA Health Forum.*

eReferences.

1. Hout M. Getting the most out of the GSS income measures. Chicago: National Opinion Research Center. 2004.

2. Hayes AF. Introduction to mediation, moderation, and conditional process analysis: A regression-based approach. Guilford Press. 2018.

© 2021 Yang H et al. *JAMA Health Forum.*
